# Comparison of Dixon Sequences for Estimation of Percent Breast Fibroglandular Tissue

**DOI:** 10.1371/journal.pone.0152152

**Published:** 2016-03-24

**Authors:** Araminta E. W. Ledger, Erica D. Scurr, Julie Hughes, Alison Macdonald, Toni Wallace, Karen Thomas, Robin Wilson, Martin O. Leach, Maria A. Schmidt

**Affiliations:** 1 CR-UK Cancer Imaging Centre, The Institute of Cancer Research and Royal Marsden NHS Foundation Trust, London, United Kingdom; 2 Department of Radiology, Royal Marsden NHS Foundation Trust, London, United Kingdom; 3 Clinical Research and Development, Royal Marsden NHS Foundation Trust, London, United Kingdom; University of Chicago, UNITED STATES

## Abstract

**Objectives:**

To evaluate sources of error in the Magnetic Resonance Imaging (MRI) measurement of percent fibroglandular tissue (%FGT) using two-point Dixon sequences for fat-water separation.

**Methods:**

Ten female volunteers (median age: 31 yrs, range: 23–50 yrs) gave informed consent following Research Ethics Committee approval. Each volunteer was scanned twice following repositioning to enable an estimation of measurement repeatability from high-resolution gradient-echo (GRE) proton-density (PD)-weighted Dixon sequences. Differences in measures of %FGT attributable to resolution, T_1_ weighting and sequence type were assessed by comparison of this Dixon sequence with low-resolution GRE PD-weighted Dixon data, and against gradient-echo (GRE) or spin-echo (SE) based T_1_-weighted Dixon datasets, respectively.

**Results:**

%FGT measurement from high-resolution PD-weighted Dixon sequences had a coefficient of repeatability of ±4.3%. There was no significant difference in %FGT between high-resolution and low-resolution PD-weighted data. Values of %FGT from GRE and SE T_1_-weighted data were strongly correlated with that derived from PD-weighted data (*r* = 0.995 and 0.96, respectively). However, both sequences exhibited higher mean %FGT by 2.9% (*p* < 0.0001) and 12.6% (*p* < 0.0001), respectively, in comparison with PD-weighted data; the increase in %FGT from the SE T_1_-weighted sequence was significantly larger at lower breast densities.

**Conclusion:**

Although measurement of %FGT at low resolution is feasible, T_1_ weighting and sequence type impact on the accuracy of Dixon-based %FGT measurements; Dixon MRI protocols for %FGT measurement should be carefully considered, particularly for longitudinal or multi-centre studies.

## Introduction

The relative amount of fibroglandular tissue (FGT) within the breast on X-Ray Mammography (XRM) is widely acknowledged to have a strong association with breast cancer risk, and is commonly known as breast density [1]. In XRM, FGT and fat appear radiologically opaque and lucent, respectively, enabling a visual assessment of mammographic breast density. In this manner, the latest edition of the Breast Imaging-Reporting and Data System (BI-RADS) assigns mammographic breast tissue composition to one of four categories, from **a**, almost entirely fatty, to **d**, extremely dense [2]. The proportion of the breast area occupied by radio-dense FGT can also be expressed as a quantitative measure of Percent Mammographic Density (PMD) [3]. A meta-analysis identified that women with ≥75% percentage density on XRM had a 4.64-fold increased risk of incident breast cancer relative to those women with <5% percent density [4]. Although quantitative PMD can be calculated [5–7], MRI has the advantage of providing a three-dimensional volumetric measurement. In addition, MRI evaluation of breast density does not use ionizing radiation and would therefore be preferred for the assessment of breast density in younger women and for longitudinal studies, where multiple breast density measurements may be made for the same individual. The manipulation of tissue relaxation times and chemical shift in MRI allows water-containing tissues to be distinguished from fat, resulting in a breast density measurement of the FGT volume in the breast and, analogous to PMD, the volume of FGT relative to the total breast volume (%FGT). Indeed, MRI-derived %FGT has been shown to correlate with PMD in high risk cohorts [8,9].

However, as yet, there is no consensus on the most appropriate MRI sequence and methodology for breast density measurement. In T_1_-weighted sequences, with and without fat suppression, FGT and fat have been separated by means of threshold-based segmentation [8–10] or the fuzzy C-means clustering algorithm [11–16]. However, both of these methods can require some subjective user interaction to define FGT by selection of the necessary threshold level or the optimal cluster number. Further, %FGT assessment is known to vary with MR sequence [17] and with the implementation of fat suppression [18,19]. Instead, Dixon fat-water separation techniques have been suggested as a more objective approach to MRI-derived density calculation since separate fat- and water-only images can be generated without user interaction [20]. The ability to accurately account for partial volume is another advantage of the Dixon technique so that imaging with lower spatial resolution can be considered [21]. However, Dixon fat- and water-only images can be generated from a number of different sequences with different contrast characteristics. Further, it must be considered that a voxel of 100% fat or 100% water will not necessarily yield the same signal intensity between the fat- and water-only images. Calculation of the volumetric water fraction therefore requires a calibration step to normalize the signal intensity which, while rarely discussed in detail within the Dixon literature, is known to vary—Boyd *et al* derived their calibration from a test object experiment [21] whilst others have used a fixed correction factor derived from the theoretical proton density difference between water and the saturated component of triglyceride molecules in adipose tissue [22,23]. Here we implement a standardized method to calculate the correction factor and assess the potential error in %FGT measurement which can arise from its miscalculation.

In this article we consider the accuracy of Dixon-based %FGT measurements. In order to distinguish between various sources of error (among these, patient positioning, breast segmentation and signal intensity correction factor), we assess %FGT repeatability and acquire, process and compare Dixon-based images with different T_1_-weighting, sequence type and resolution in a group of volunteers. With this approach we identify causes of error and thereby provide guidance on MRI protocols for Dixon-based %FGT measurements.

## Materials & Methods

### Study Cohort

Research Ethics Committee (UK NHS HRA) approval was granted (NRES Committee London-Chelsea; Reference No. 1406, Date: 18-06-1997) and written informed consent was obtained from all subjects in this study. Ten healthy female volunteers (median age: 31 yrs, range: 23–50 yrs) were recruited; a broad age band was chosen to confer a wider range of breast density values. Each volunteer underwent two MRI breast examinations without contrast to enable an estimation of measurement repeatability. The two scans were performed on the same day to exclude the influence of hormonal cycle changes.

### Image Acquisition

Breast images were acquired at 1.5T (MAGNETOM Aera, Siemens AG, Germany) using the Sentinelle breast coil with Variable Coil Geometry (Invivo International Ltd, Netherlands). Prior to each scan, a different radiographer reconfigured the lateral, moveable, breast coils from a standard neutral location and the volunteer was positioned with the nipple aligned to the coil centre. During the first examination, volunteers were scanned with forward arm extension, but were encouraged to reposition the arms alongside the body for the repeat scan should they have experienced any discomfort. Three different Dixon sequences were acquired in the first MRI examination of each volunteer. Two high-resolution, two-point 3D gradient-echo (GRE) based Dixon sequences were acquired with PD- and T_1_-weighting (Repetition Time (TR) = 7.34 ms, Echo Time (TE) = 4.77/2.39 ms, Voxel Size = 1.3×1.3×1.0 mm^3^, Receiver Bandwidth = 390 Hz/Px, Parallel Imaging Factor = 2 (GRAPPA), Acquisition Time = 2 min 40 s, Flip Angle (FA) = 4° and 25°, respectively), denoted as HR GRE PD and HR GRE T1, respectively. A further low-resolution 2D spin-echo (SE) based T_1_-weighted two-point Dixon sequence with a longer echo train (TR = 500 ms, TE = 12 ms, in-phase and out-of-phase echoes, In-Plane Resolution = 0.8×0.8 mm^2^, Slice Thickness = 7.0 mm, Echo Spacing = 12.2 ms, Receiver Bandwidth = 275 Hz/Px, Parallel Imaging Factor = 2 (GRAPPA), Echo Train Length = 8, Acquisition Time = 4 min 59 s) was also acquired, denoted LR SE T1. In the second MRI examination for each volunteer, a repeat high-resolution gradient-echo PD-weighted sequence with identical parameters was acquired to enable a repeatability measurement, denoted as HR GRE PD (R).

### Data Analysis

#### Post-acquisition processing

From the first MRI examination, two further low-resolution GRE Dixon datasets were generated from the high-resolution GRE sequences using Siemens Multi-Planar Reformatting to match the low resolution SE T_1_-weighted sequence, denoted as LR GRE PD and LR GRE T1, respectively. In total, for each volunteer, this resulted in five datasets covering the same volume for comparison within the first MRI examination: a high-resolution GRE PD-weighted reference sequence (HR GRE PD), a high-resolution GRE T_1_-weighted sequence (HR GRE T1), a low-resolution GRE PD-weighted dataset (LR GRE PD), a low-resolution GRE T_1_-weighted dataset (LR GRE T1) and a low-resolution SE T_1_-weighted sequence (LR SE T1).

#### Breast segmentation

Semi-automated breast volume segmentation was performed by a researcher (AEWL) on the in-phase GRE PD-weighted datasets (at high and low resolution) using in-house software (IDL 8.3, ITTVIS, Boulder, USA). The external contour of the breast volume (retaining the skin) was delineated by a combination of manual signal intensity thresholding and erosion of the background noise (standard erosion implementation in the IDL package library). For each breast, a manual straight coronal cut was performed at the most anterior position of the pectoral muscle to define the posterior limit [8]. This approach automatically defined the superior and inferior limits of the breast and generated three volumetric segmentation masks for each breast: high and low resolution masks from the first MRI examination and a further repeat high resolution mask from the second MRI examination.

#### Signal intensity correction factor

**Fig 1:**
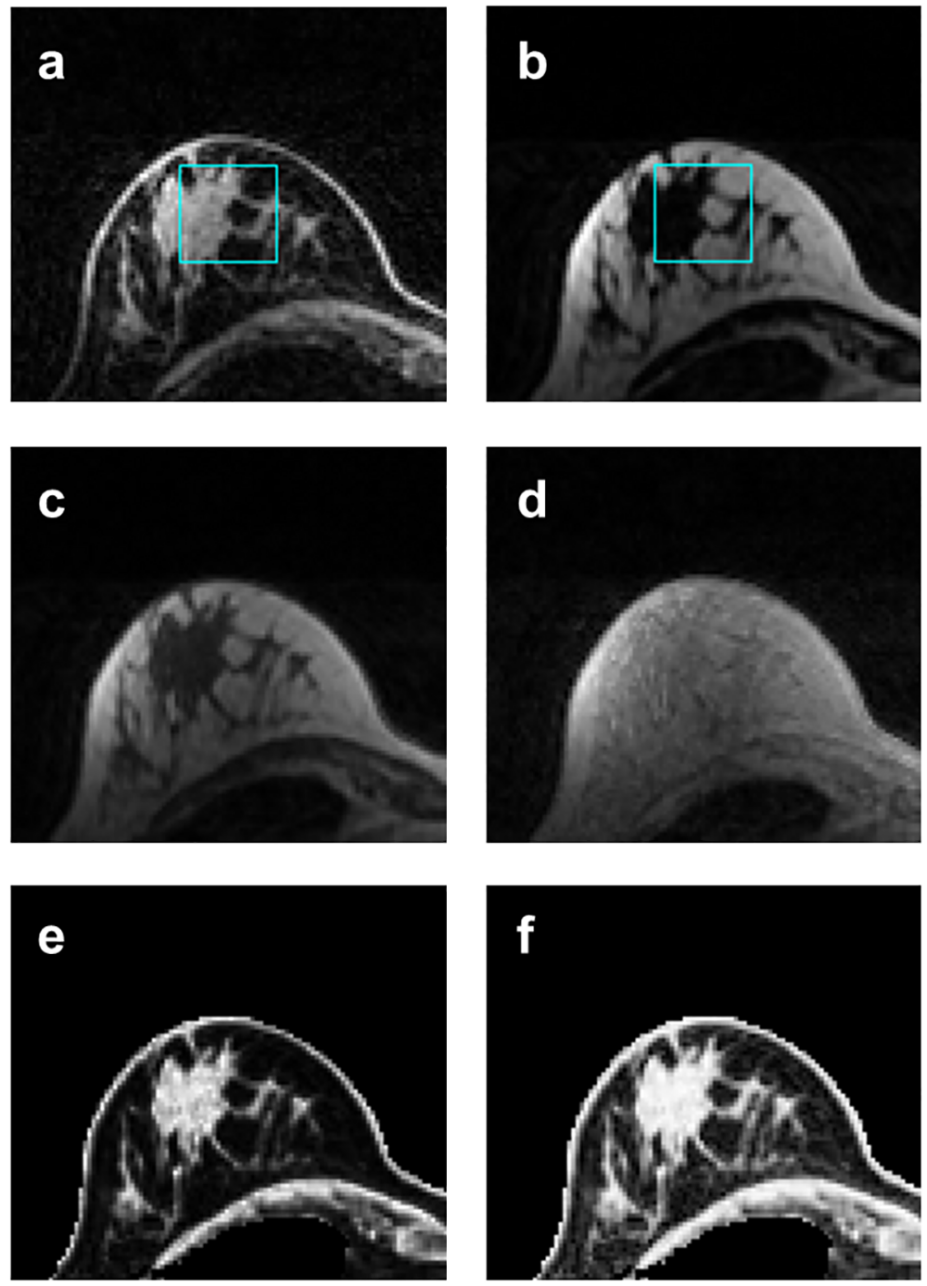
High resolution GRE T_1_-weighted Dixon breast images of the right breast in a 31 year old volunteer, showing the effect of signal intensity correction. A signal intensity correction factor was derived from the maximum water and fat signal intensities within a centrally located region of interest (ROI) on the Water- and Fat-only images (**a** and **b**, respectively, each scaled to their maximum signal intensity) and was applied to the Water-only image. The |Water + Fat| and Water Fraction images prior to signal intensity correction are shown in **c** and **e**, respectively. The equivalent images following signal intensity correction are displayed in **d** and **f**, respectively; **d** shows clearly the normalized contributions of water and fat following correction of the Water-only image. Calculated %FGT was found to be 34.4%.

For the fat- and water-only images of each Dixon dataset, the maximum signal intensity was measured from a standardized 30x30 mm^2^ region of interest (ROI) in the centre of each breast (to minimize possible coil sensitivity effects) in a central slice containing fat and water (Fig 1). The relative magnitudes of the maximum fat and water signal intensities (F_max_ and W_max_, respectively) were then used to calculate a correction factor for the water-only image of each Dixon dataset (as the ratio of F_max_/W_max_). For every voxel within the volumetric segmentation mask, the water fraction, *WF*, was calculated as

$$WF_{ijk}=\;\frac{W_{ijk}^{c}}{W_{ijk}^{c}+F_{ijk}}$$

where *W*^*c*^ and *F* are the corrected water and fat signal intensities at each voxel location (i, j, k). Dixon fat/water separation techniques to measure breast density assume that water-containing tissues represent the fibroglandular content of the breast. The summation of voxel water fractions multiplied by the voxel size therefore yielded the total volume of fibroglandular tissue (FGT) whilst the percentage of FGT volume relative to the volume of the segmentation mask resulted in a measurement of %FGT for each breast in each Dixon dataset.

### Investigation into sources of error

In this study, the high resolution GRE PD-weighted datasets were assumed to provide the most accurate assessment of %FGT since the variation in signal intensity across the fat- and water-only images in this sequence is minimal, with the least influence of tissue characteristics. As such, a correction factor can be easily estimated via the method above to transform the image intensity into a surrogate for the volume occupied by water and fat within the breast. The HR GRE PD datasets therefore provided the reference standard. Individual comparisons between select Dixon datasets were then made to separate the sources of discrepancy between %FGT measurements from different sequence alterations.

#### Repeatability

Comparison of FGT volume, total breast volume and %FGT between the HR GRE PD and HR GRE PD (R) datasets enabled a measure of repeatability which accounted for changes in patient positioning, coil profile and intra-operator variability in the breast volume segmentation.

#### Resolution and T_1_ weighting

Using datasets from the first MRI examination, the %FGT calculated from each Dixon dataset under investigation was compared with that derived from the reference GRE PD weighted data. To isolate each potential source of error (T_1_ weighting/sequence type or resolution), the same breast volume segmentation at either high or low resolution was applied to the datasets following a rigid registration based on a mutual information algorithm [24]. Thus, a comparison of the HR GRE T1 and HR GRE PD datasets provided information on the effect of T_1_ versus PD weighting on the %FGT measurement, since these datasets differed in contrast but not in pixel size. A comparison between LR GRE T1 and LR SE T1 datasets and the LR GRE PD dataset provided information on the differences in %FGT conferred by T_1_ weighting and by sequence type. In datasets with the same T_1_ weighting, such as between the HR GRE PD and LR GRE PD datasets, comparison provided information on the effect of resolution on the %FGT measurement.

#### Investigation of applied signal intensity correction factor

The repeatability in calculated signal intensity correction factor between the repeat HR GRE PD Dixon datasets was evaluated, and any difference in correction factor with spatial resolution was assessed. Measurements of %FGT from the reference sequence (HR GRE PD) were used to select two volunteers with high and low breast density, respectively. Using the right breast of these volunteers, the corrected Dixon water fraction for the LR GRE T1 and LR SE T1 datasets were plotted against the LR GRE PD data on a voxel by voxel basis. To assess the potential error conferred on measurement of %FGT by the signal intensity correction factor, the applied correction factor for each subject at each T_1_ weighting was over- and under-estimated by 15% and the voxel-by-voxel water fraction recalculated.

### Statistical Analysis

Coil positioning, breast volume (and chest wall) segmentation and signal intensity correction were performed separately and independently for left and right breasts in each image dataset. Statistical testing therefore treated right and left breast measures independently despite originating from the same examination, resulting in a sample size of 20 for each measurement parameter. Data were assessed visually using boxplots to inspect for deviations from the normal distribution. Differences in FGT volume, total breast volume, %FGT and signal intensity correction factor between left and right breasts were evaluated using the paired Student’s *t*-test.

#### Repeatability

Measurement repeatability of FGT volume, total breast volume and %FGT between HR GRE PD and HR GRE PD (R) datasets were evaluated using Bland-Altman statistics, where the coefficient of repeatability, *r*, was defined as ±1.96 *σ*_*d*_ where *σ*_*d*_ represents the sample standard deviation of the differences between the paired measures. The coefficient of variation, CoV, which quantifies the random errors in a single measurement, was calculated as $\sigma_{d}/\left(\sqrt{2}\bar{x}\right)*100\%$ where $\bar{x}$ represents the population mean of the means between the paired measures.

#### Resolution and T_1_ weighting

Values of %FGT for different pairs of sequences were correlated using the Pearson product-moment correlation coefficient. Measured values of %FGT were compared within each breast between datasets at varying spatial resolution, T_1_ weighting and sequence type using analysis of variance (ANOVA) together with selected comparisons between particular sequence groups using the paired Student’s *t*-test (two-sided α = 0.05). No correction was made for multiple post-hoc comparisons so the family-wise error rate may be higher than 0.05. Differences in FGT volume and total breast volume between HR and LR GRE PD datasets were also assessed using the paired Student’s *t*-test (two-sided α = 0.05).

#### Investigation of applied signal intensity correction factor

The repeatability of the applied signal intensity correction factor between the repeat HR GRE PD sequences was evaluated using Bland-Altman statistics. The signal intensity correction factors applied to high and low resolution GRE PD-weighted data were compared using the paired Student’s *t*-test (two-sided α = 0.05). Correlations between voxel-by-voxel water fractions for the low resolution data of two subjects were assessed using the Pearson product-moment correlation coefficient.

## Results

Table 1 displays the means and corresponding sample standard deviations of FGT volume, total breast volume and %FGT in the 10 volunteers for the five Dixon datasets from the first MRI examination together with the reference dataset from the repeat MRI examination, HR GRE PD (R); also shown are the corresponding mean and sample standard deviations of the calculated signal intensity correction factors applied to each sequence.

**Table 1:** Mean and corresponding sample standard deviations for FGT volume, total breast volume and %FGT measured from the different Dixon datasets in 10 volunteers. Also shown are the mean and corresponding sample standard deviations of the calculated signal intensity correction factors applied to each sequence.

Sequence Type	HR GRE PD	HR GRE PD (R)	HR GRE T1	LR GRE PD	LR GRE T1	LR SE T1
**FGT Volume [cm**^**3**^**]**	143.7 ± 63.5	135.2 ± 56.2	157.6 ± 69.5	134.2 ± 57.8	153.7 ± 68.8	207.3 ± 109.4
**Total Breast Volume [cm**^**3**^**]**	492.6 ± 299.2	482.6 ± 296.2	492.6 ± 299.2	484.2 ± 298.6	484.2 ± 298.6	484.2 ± 298.6
**%FGT**	36.3 ± 16.5	35.4 ± 16.2	39.2 ± 16.3	35.2 ± 16.5	39.1 ± 16.1	47.8 ± 11.6
**SI correction factor**	1.21 ± 0.16	1.07 ± 0.18	3.27 ± 0.38	1.14 ± 0.22	3.36 ± 0.68	2.91 ± 0.63

FGT = Fibroglandular tissue, HR = High Resolution, LR = Low Resolution, GRE = Gradient Echo, SE = Spin Echo, PD = Proton Density, SI = Signal Intensity, (R) indicates a repeat dataset used to generate repeatability measurements.

Analysis results by individual volunteer, laterality and sequence type may be found within S1 Table. Representative HR GRE Dixon images from the right breast of one volunteer are shown in Fig 1. There were no significant differences in FGT volume, total breast volume, %FGT and signal intensity correction factor between left and right breasts in any Dixon dataset.

### Investigation into sources of error

#### Repeatability

**Fig 2:**
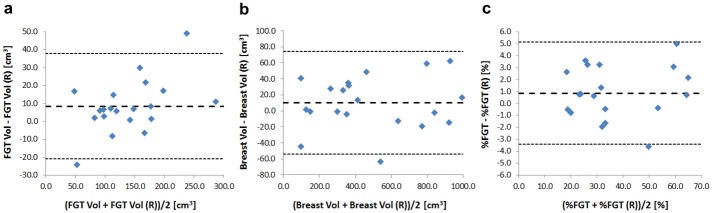
Bland-Altman plots for repeat high-resolution GRE PD-weighted measurements (HR GRE PD & HR GRE PD (R), respectively). a) FGT volume [cm^3^]; b) Total breast volume [cm^3^] and c) %FGT [%]. Mean differences and limits of agreement are represented by the central and outer dashed lines, respectively.

Although a significant difference in mean FGT volume of 8.5 cm^3^ (*p* = 0.02) was detected between the HR GRE PD and HR GRE PD (R) datasets, no significant differences were observed in total breast volume and %FGT. Coefficients of repeatability in FGT volume, total breast volume and %FGT between the two HR GRE PD datasets were found to be ±29.4 cm^3^, ±64.1 cm^3^ and ±4.3%, respectively (Fig 2). Coefficients of variation in FGT volume, total breast volume and %FGT between the two HR GRE PD sequences were calculated as 7.6%, 4.7% and 4.3%, respectively.

#### Resolution and T_1_ weighting

**Fig 3:**
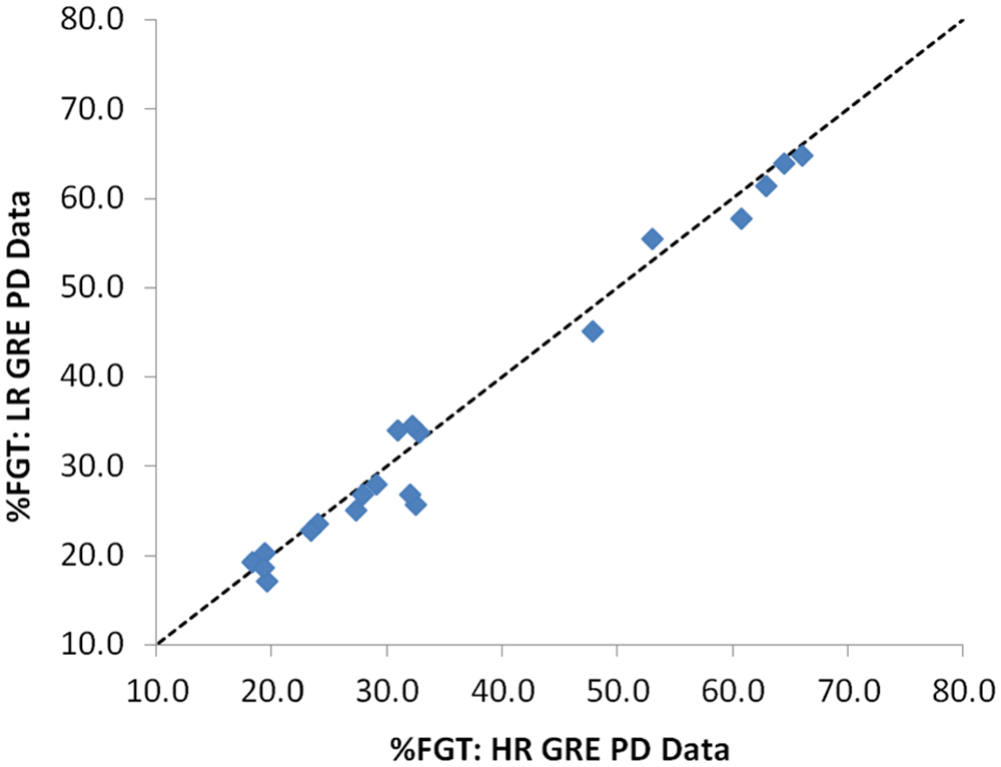
Correlation between %FGT measurements from high and low resolution GRE PD-weighted data.

**Fig 4:**
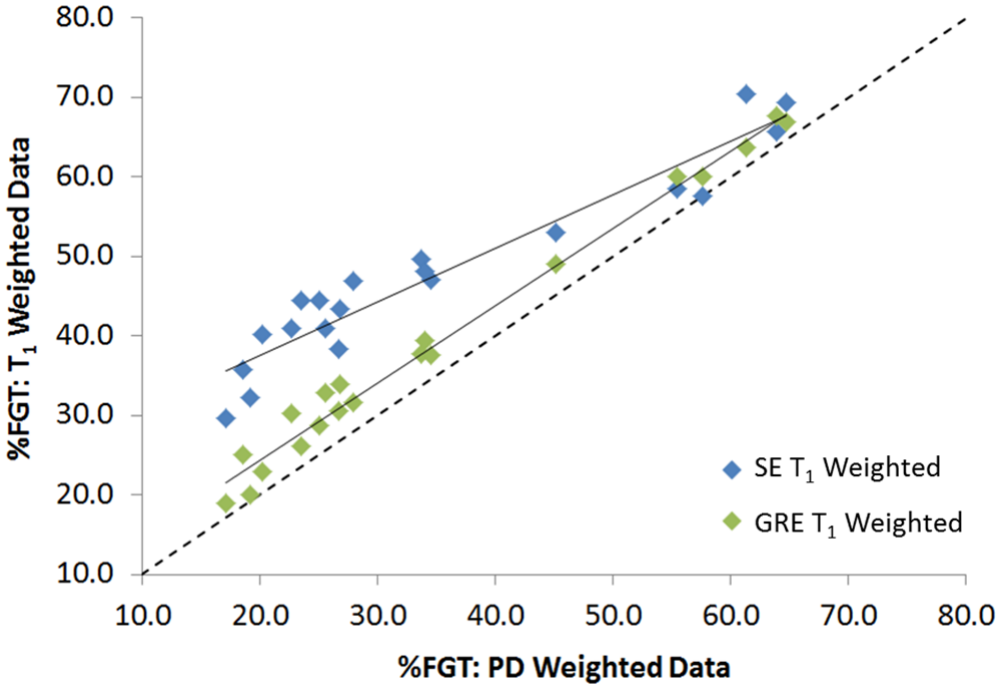
Correlation between low resolution %FGT measurements from GRE and SE T_1_-weighted data *vs*. GRE PD-weighted data.

Sequence alterations resulted in statistically significant differences in %FGT (*p* < 0.0001). %FGT derived from high and low resolution GRE PD data were strongly correlated (*r* = 0.99) with no significant difference in mean %FGT (*p* = 0.06) (Fig 3). %FGT measurements from GRE T_1_-weighted data were strongly correlated with those from GRE PD-weighted data at both high and low resolution (*r* = 0.995 and *r* = 0.99, respectively) but exhibited higher mean %FGT in comparison with GRE PD-weighted data (+2.9%, *p* < 0.0001 and +3.9%, *p* < 0.0001, respectively). LR SE T1%FGT values were strongly correlated with LR GRE PD values (*r* = 0.96), but mean %FGT from LR SE T1 data was 12.6% higher (*p* < 0.0001) than that from GRE PD-weighted data. This increase in %FGT was significantly larger at lower breast densities (Fig 4).

#### Investigation of applied signal intensity correction factor

Between the repeat HR GRE PD sequences, a significant difference in mean correction factor of 0.13 was detected (*p* = 0.006), with a coefficient of repeatability of ±0.38 and coefficient of variation calculated at 12.0%. There was no significant difference in mean correction factor applied to the high and low resolution GRE PD-weighted sequences.

**Fig 5:**
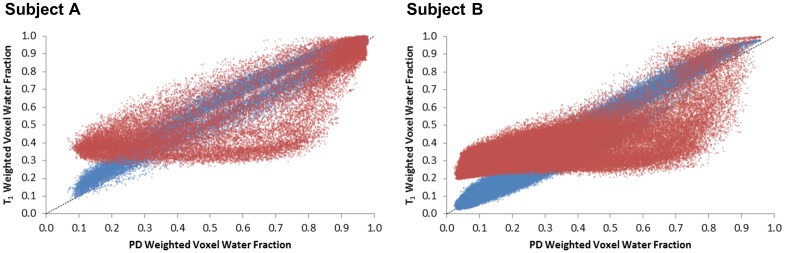
Low resolution voxel water fractions in corrected GRE and SE T_1_-weighted data *vs*. corrected GRE PD-weighted data (*blue and red*, *respectively*) for the right breast of two subjects. There is an approximately linear relationship between corrected GRE T1 and PD data, but a pronounced deviation of corrected SE T1 voxel water fractions from lower GRE PD values; A) 26 year old volunteer with 64.6% FGT and B) 50 year old volunteer with 18.4% FGT.

**Fig 6:**
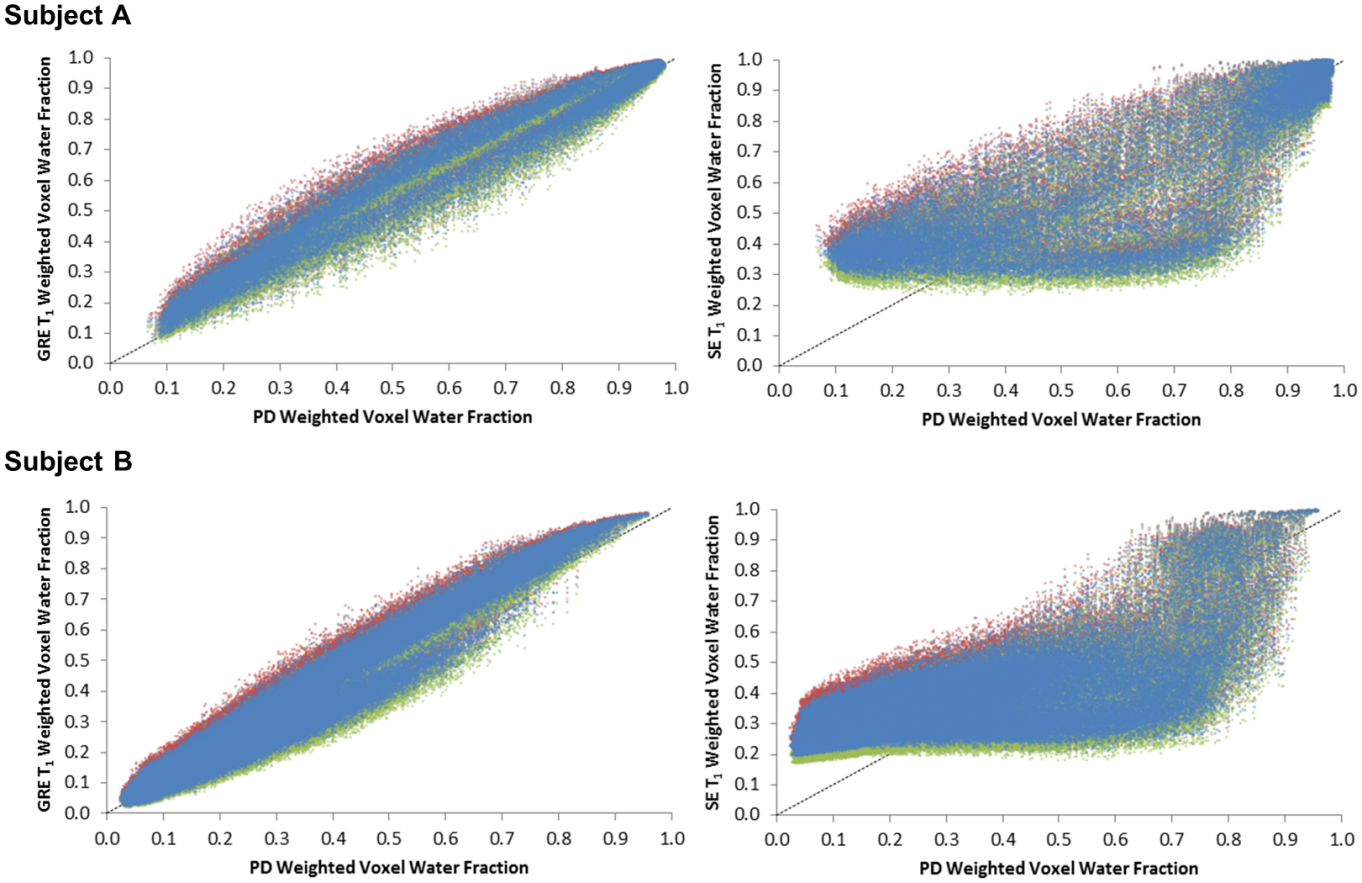
Low resolution voxel water fractions for corrected GRE and SE T_1_-weighted data (*left and right*, *respectively*) *vs*. corrected GRE PD-weighted data showing the minimal perturbation in water fraction values caused when the original signal correction (*blue*) is altered by +15% and -15% (*red and green*, *respectively*) for the right breast of two subjects. The relationship between SE T_1_-weighted data and GRE PD-weighted data is not linear; A) 26 year old volunteer with 64.6% FGT and B) 50 year old volunteer with 18.4% FGT.

**Fig 7:**
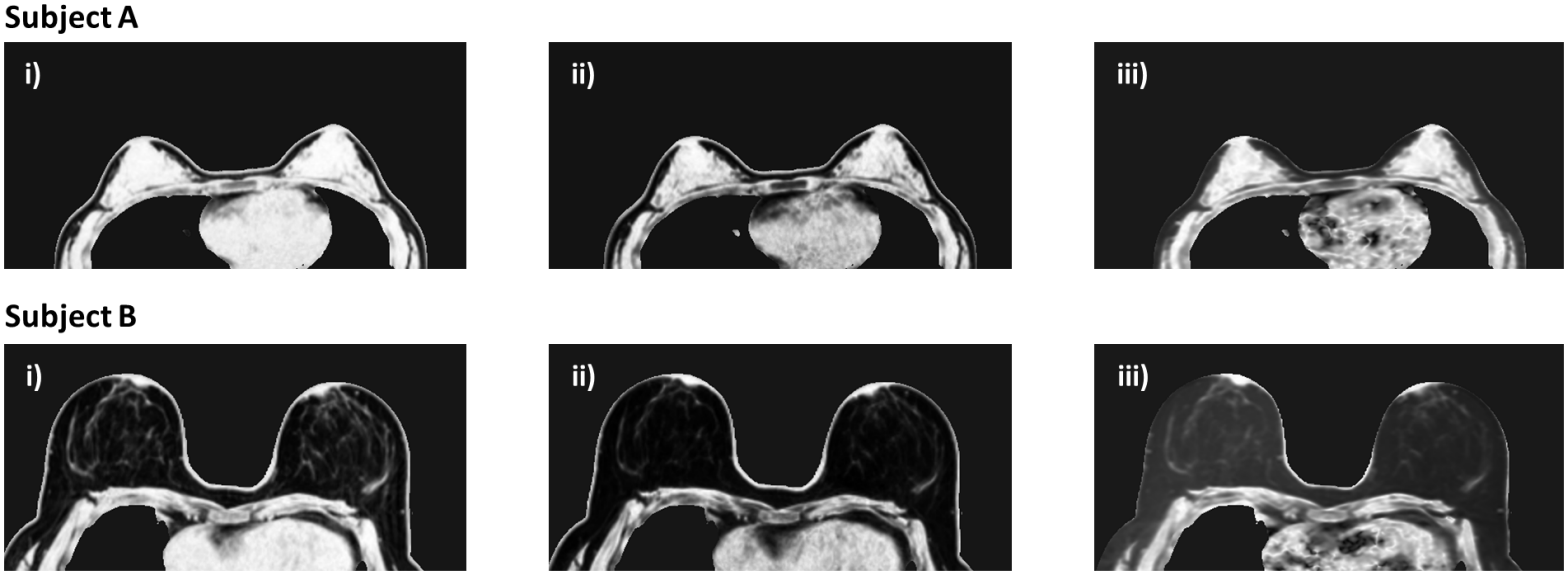
Low resolution water fraction images prior to signal correction for two subjects. A) a 26 year old volunteer with 64.6% FGT and B) a 50 year old volunteer with 18.4% FGT. GRE PD, GRE T_1_ and SE T_1_-weighted images are shown in i), ii) and iii), respectively—the water fraction values within the fat are much higher in the SE T_1_-weighted datasets.

A 26-year-old and a 50-year-old volunteer with %FGT of 64.6% and 18.4% in the right breast, respectively, were selected for further analysis of the applied signal intensity correction factor: denoted Subjects A and B, respectively. A high correlation was found for both subjects between the corrected LR GRE T1 and corrected LR GRE PD voxel water fractions (*r* = 0.99 and *r* = 0.98 for Subjects A and B, respectively). However, the correlation was lower between the corrected LR SE T1 and LR GRE PD voxel water fractions for either subject (*r* = 0.86 and *r* = 0.74, respectively) (Fig 5). For Subject A, deliberately over- or under-estimating the correction factor by 15% altered the calculation of %FGT by +2.0 / ‒2.4% for the LR GRE T1 dataset and by +2.2 / ‒2.6% for the LR SE T1 dataset. For Subject B, the same deliberate introduction of a ±15% error in the correction factor resulted in a +1.8 / ‒2.0% alteration in %FGT in the LR GRE T1 dataset and a +3.0% / ‒3.3% difference in %FGT in the LR SE T1 dataset. However, the overall trends in correlation with the LR GRE PD %FGT data remained unaltered (Fig 6). Low-resolution GRE PD, GRE T_1_ and SE T_1_-weighted water fraction images for each subject prior to signal intensity correction are displayed in Fig 7.

## Discussion

This study considered the potential sources of error in %FGT measurement induced by sequence alterations in two-point Dixon fat-water separation techniques, and the impact of these errors on the outcome of %FGT measurement for MR-derived breast density studies.

%FGT measurement from HR GRE PD sequences was highly repeatable; we report a coefficient of repeatability of ±4.3% and a coefficient of variation of 4.3%, accounting for changes in both subject and coil position as well as intra-operator variability in data-processing. Although repeatability metrics vary widely in the literature, these results compare favourably with studies which assessed repeat %FGT measurement after subject repositioning, including those which utilised a fuzzy C-means clustering segmentation [13,20,25], and a further study which assigned FGT in Dixon imaging on the basis of automated signal intensity histogram thresholding [26]. The significant difference in signal intensity correction factor and FGT volume observed between the repeat HR GRE PD datasets may be derived from the change in volunteer arm positioning, although this discrepancy did not translate into a significant difference between repeat %FGT measurements. In addition, this study utilized a straight coronal cut at the most anterior position of the pectoral muscle to segment the breast volume [8]. This approach will have also contributed to the observed measurement error due to the differences in chest wall position between the volunteer datasets.

This study suggests that low spatial resolution PD-weighted Dixon sequences could reasonably be used to assess breast density; presuming similar repeatability at high and low resolution, low resolution sequences have the added advantages of a reduction in scan time, an increase in signal-to-noise ratio (SNR) and fewer slices for analysis. This result highlights the ability of Dixon methods to account for partial volume effects, although the impact of resolution on %FGT measurement may be influenced by breast volume segmentation technique.

Despite an applied signal correction, both GRE and SE T_1_-weighted data exhibited higher mean %FGT than PD-weighted data. Although this difference was lower than the coefficient of repeatability in the GRE datasets, that derived from the spin-echo T_1_-weighted dataset (12.6%) was particularly substantial and increased considerably at lower breast densities. This also reduced the range of volunteer %FGT values in the LR SE T1 dataset (40.7%) relative to the LR GRE PD or LR GRE T1 datasets (47.6% and 48.7%, respectively).

Although an approximately linear relationship was apparent between the corrected LR GRE T1 and LR GRE PD voxel water fractions, the corrected LR SE T1 voxel water fractions strongly deviated from lower LR GRE PD values in subjects with both high and low breast density (Subjects A and B, respectively, Fig 5). The increasing deviation of %FGT at lower breast densities in the LR SE T1 data was clearly derived from the effect of the relative number of voxels at lower water fractions on the total voxel-by-voxel summation. Recalculating the LR GRE T1 and LR SE T1 voxel water fractions for Subjects A and B with the correction factor altered by ±15% only resulted in marginal %FGT differences, below the limits set by the coefficient of repeatability, and did not change the overall relationships (Fig 6). This established that an error in determination of the correction factor could not account for the observed differences in mean %FGT.

Although the LR SE T1 dataset featured T_1_-weighted values of TR and TE, the long echo train may have introduced some T_2_-weighting to the sequence. Indeed, the uncorrected water fraction images for Subjects A and B in Fig 7 reveal the different image contrast of the LR SE T1 dataset at lower water fractions which could not be rectified by the correction factor. The most likely causes of this discrepancy have been discussed in the Dixon fat-water separation literature: the fat spectrum may be poorly approximated by a single peak [23,27] and there are differences between the real and complex reconstructions of the fat and water images [28]. Yu *et al*. and Eggers *et al*. highlight differences affecting voxels where the water fraction is low, coinciding exactly with the problematic voxels in our measurement [27,28]. This suggests that different Dixon fat-water separation algorithms may perform differently, adding a degree of complexity to the standardization of multi-centre studies. However, comparison of the two-point fat-water separation technique we employed with other Dixon-based approaches was beyond the purpose of this study [17,29–31].

A comparison of %FGT measurement using Dixon sequences with other segmentation approaches was also outside the scope of this work. However, a recent study identified significant differences between %FGT derived from a three-point Dixon technique and that from the fuzzy C-means clustering segmentation of a T_1_-weighted sequence—with particularly pronounced deviation at lower breast densities [20]. In light of the errors identified here, it would be interesting to reassess the relationship between the two approaches.

Our study was limited to sequence assessment from a single 1.5T MRI scanner whilst Dixon implementation may vary between field strengths, MRI systems and vendors. In addition, we employed only a two-point Dixon fat/water separation technique, whilst a three-point technique is acknowledged to provide a %FGT calculation which better accounts for phase differences arising from B_0_ field inhomogeneity [32]. Further, we did not employ any signal intensity bias field correction to account for B_1_ field inhomogeneity [33]. In the absence of an accepted gold-standard for Dixon fat-water separation sequences, this study assumed that HR GRE PD sequences would provide the most accurate measurement of %FGT. An assessment of inter-observer variation (reproducibility) was beyond the range of this study, which looked to assess the impact of sequence alterations on calculation of %FGT, but would be highly relevant to establish use of our interactive-thresholding technique in clinical practice or to compare segmentation techniques.

Dixon fat/water separation techniques have been considered a more objective approach to MR-derived breast density assessment with reduced user interaction [20]. Several studies have used a Dixon %FGT measurement to further investigate the relationship between breast density and breast cancer risk, using sequences with varying resolution and image weighting [21,34]. However, this study has demonstrated that the selection of T_1_ weighting and sequence type (with standardized methods for breast volume segmentation, signal correction and %FGT calculation) can strongly influence the measurement of %FGT. These errors could potentially influence the study of breast cancer risk factors and could mask density changes in longitudinal studies. In addition, our results are highly relevant to the comparison of breast density studies from different centres and to the analysis of multi-centre trials.

In conclusion, our study demonstrates that Dixon-based measurements of %FGT are highly repeatable and can account for partial volume effects: %FGT can be accurately derived from low resolution Dixon images. However, the selection of MRI protocols for Dixon measurement of %FGT should be carefully considered, as it may not be possible to compensate for changes in image contrast within the fat and water images across the full range of %FGT values. In the wider field, further work is needed to standardize Dixon protocols and to ensure the clinical accuracy of breast density measured via this technique.

## Supporting Information

S1 Table FGT volume, total breast volume and %FGT from Dixon datasets at different spatial resolutions and with different T_1_ weighting in 10 volunteers (right & left measures). (DOCX)
