# Supplementary material for: Comparison of Dixon Sequences for Estimation of Percent Breast Fibroglandular Tissue
Source: PLoS One. 2016 Mar 24;11(3):e0152152. doi: 10.1371/journal.pone.0152152 (PMC4806997; doi:10.1371/journal.pone.0152152)
Supplement: S1 Table — (DOCX) [file pone.0152152.s001.docx]

**Supporting Information**

**S1 Table.** **FGT volume, total breast volume and %FGT from Dixon datasets at different spatial resolutions and with different image weighting in 10 volunteers (right & left measures)**

|  | **Volunteer & Laterality** | | | | | | | | | | | | | | | | | | | |  |
| --- | --- | --- | --- | --- | --- | --- | --- | --- | --- | --- | --- | --- | --- | --- | --- | --- | --- | --- | --- | --- | --- |
| **Sequence Type** | **1-R** | **1-L** | **2-R** | **2-L** | **3-R** | **3-L** | **4-R** | **4-L** | **5-R** | **5-L** | **6-R** | **6-L** | **7-R** | **7-L** | **8-R** | **8-L** | **9-R** | **9-L** | **10-R** | **10-L** |  |
| **HR GRE T1 FGT [cm^3^]** | 121.7 | 113.9 | 128.1 | 138.6 | 214.4 | 153.7 | 317.4 | 289.8 | 130.6 | 113.1 | 85.3 | 103.3 | 178.8 | 184.3 | 162.6 | 185.8 | 217.6 | 207.9 | 44.1 | 61.9 |  |
| **HR GRE PD FGT [cm^3^]** | 94.5 | 100.9 | 113.4 | 121.9 | 206.4 | 142.5 | 292.1 | 262.5 | 121.7 | 108.4 | 83.1 | 99.4 | 173.9 | 181.4 | 152.1 | 179.7 | 179.0 | 163.2 | 40.3 | 57.0 |  |
| **Breast Volume [cm^3^]** | 486.0 | 420.1 | 344.8 | 377.7 | 633.0 | 508.7 | 1002.8 | 958.2 | 379.2 | 350.0 | 128.6 | 150.4 | 276.3 | 298.3 | 828.0 | 914.5 | 760.8 | 840.4 | 75.8 | 119.0 |  |
| **HR GRE T1 %FGT** | 25.0 | 27.1 | 37.1 | 36.7 | 33.9 | 30.2 | 31.7 | 30.2 | 34.4 | 32.3 | 66.3 | 68.7 | 64.7 | 61.8 | 19.6 | 20.3 | 28.6 | 24.7 | 58.2 | 52.1 |  |
| **HR GRE PD %FGT** | 19.4 | 24.0 | 32.9 | 32.3 | 32.6 | 28.0 | 29.1 | 27.4 | 32.1 | 31.0 | 64.6 | 66.1 | 62.9 | 60.8 | 18.4 | 19.7 | 23.5 | 19.4 | 53.1 | 47.9 |  |
|  |  |  |  |  |  |  |  |  |  |  |  |  |  |  |  |  |  |  |  |  |  |
| **LR GRE T1 FGT [cm^3^]** | 111.5 | 112.1 | 134.5 | 134.5 | 201.9 | 147.0 | 313.6 | 264.2 | 119.6 | 130.4 | 78.3 | 97.1 | 182.6 | 175.5 | 165.3 | 171.0 | 231.1 | 212.9 | 33.9 | 56.6 |  |
| **LR SE T1 FGT [cm^3^]** | 196.1 | 190.7 | 177.0 | 168.2 | 251.6 | 185.1 | 465.1 | 410.1 | 152.9 | 159.1 | 76.2 | 100.7 | 201.8 | 168.3 | 266.6 | 268.3 | 311.6 | 302.9 | 33.1 | 61.1 |  |
| **LR GRE PD FGT [cm^3^]** | 98.3 | 100.8 | 120.3 | 123.6 | 157.2 | 128.9 | 277.0 | 230.7 | 94.6 | 112.6 | 74.0 | 94.0 | 176.0 | 168.6 | 158.5 | 154.8 | 172.9 | 157.5 | 31.3 | 52.1 |  |
| **Breast Volume [cm^3^]** | 487.6 | 429.6 | 356.7 | 358.1 | 614.3 | 482.3 | 993.2 | 922.0 | 352.8 | 331.0 | 115.9 | 145.3 | 286.8 | 292.4 | 828.2 | 905.8 | 762.3 | 848.2 | 56.6 | 115.5 |  |
| **LR GRE T1 %FGT** | 22.9 | 26.1 | 37.7 | 37.6 | 32.9 | 30.5 | 31.6 | 28.6 | 33.9 | 39.4 | 67.6 | 66.8 | 63.7 | 60.0 | 20.0 | 18.9 | 30.3 | 25.1 | 60.0 | 49.0 |  |
| **LR SE T1 %FGT** | 40.2 | 44.4 | 49.6 | 47.0 | 41.0 | 38.4 | 46.8 | 44.5 | 43.3 | 48.1 | 65.7 | 69.3 | 70.3 | 57.6 | 32.2 | 29.6 | 40.9 | 35.7 | 58.5 | 52.9 |  |
| **LR GRE PD %FGT** | 20.2 | 23.5 | 33.7 | 34.5 | 25.6 | 26.7 | 27.9 | 25.0 | 26.8 | 34.0 | 63.9 | 64.7 | 61.4 | 57.6 | 19.1 | 17.1 | 22.7 | 18.6 | 55.4 | 45.1 |  |
|  |  |  |  |  |  |  |  |  |  |  |  |  |  |  |  |  |  |  |  |  |  |
| **HR GRE PD (R) FGT [cm^3^]** | 88.5 | 94.4 | 106.3 | 116.2 | 189.4 | 141.8 | 281.0 | 213.3 | 106.9 | 116.6 | 81.1 | 96.6 | 143.9 | 172.8 | 145.1 | 157.8 | 177.6 | 169.6 | 64.5 | 40.1 |  |
| **Breast Volume [cm^3^]** | 437.2 | 406.6 | 318.7 | 342.7 | 645.5 | 572.5 | 986.4 | 896.1 | 347.7 | 354.5 | 126.9 | 151.1 | 248.3 | 299.3 | 769.1 | 928.8 | 780.0 | 842.6 | 120.6 | 78.0 |  |
| **HR GRE PR (R) %FGT** | 20.2 | 23.2 | 33.4 | 33.9 | 29.3 | 24.8 | 28.5 | 23.8 | 30.8 | 32.9 | 63.9 | 63.9 | 57.9 | 57.7 | 18.9 | 17.0 | 22.8 | 20.1 | 53.5 | 51.5 |  |
|  |  |  |  |  |  |  |  |  |  |  |  |  |  |  |  |  |  |  |  |  |  |
